# Synchronous Occurrence of Guillain-Barre Syndrome and Transverse Myelitis of Unknown Etiology in an Adolescent

**DOI:** 10.7759/cureus.9645

**Published:** 2020-08-10

**Authors:** Ankit Agarwal, Adriana Fernandez Bowman

**Affiliations:** 1 Pediatrics, Ascension Sacred Heart Hospital, Pensacola, USA; 2 Pediatric Medicine, University of Florida College of Medicine, Pensacola, USA

**Keywords:** guillain-barre syndrome, acute transverse myelitis, synchronous occurrence, unknown etiology

## Abstract

Synchronous occurrence of Guillain-Barre syndrome (GBS) and acute transverse myelitis (ATM) happens very rarely in childhood. Only a few cases of these conditions occurring simultaneously have been reported and represent a diagnostic challenge. We describe a case of a 17-year-old male presenting with acute onset of bilateral symmetrical numbness and tingling starting in the feet that rapidly ascended to the legs with associated motor weakness, associated with a sensory level and urinary retention. Albuminocytologic dissociation on cerebrospinal fluid (CSF) analysis was consistent with GBS. MRI spine revealed an area of increased T2 signal involving the dorsal aspect of the left side of the cord at the C7 level. The patient was treated with IV methylprednisolone and IV immunoglobulin with significant improvement. This report emphasizes the rarity of this synchronous occurrence in children and the need for further reports to understand the mechanism and better treatment approaches.

## Introduction

Guillain-Barre syndrome (GBS) and acute transverse myelitis (ATM) are both autoimmune diseases with many differences in histologic and pathologic evidence [1]. Pediatric ATM is a demyelinating immune-mediated central nervous system disorder [2]. The first symptom in patients with ATM can be back pain, followed by bilateral limb paralysis, motor and sensory deficits or bladder/bowel dysfunction [1, 3-4]. Most children develop urinary retention. It typically manifests itself over a period of hours to one week. ATM is either caused by myelitis due to direct infection of the spinal cord or autoimmune processes [5]. GBS is characterized by rapidly progressive symmetrical weakness of the limbs with hyporeflexia or areflexia; sensory disturbances and cranial nerve deficits occur in some patients [6]. GBS typically occurs after an infectious disease in which the immune response generates antibodies that are directed against myelin proteins of peripheral nerves [6-7].

The synchronous occurrence should be suspected in patients (1) presenting with signs of areflexia or hyporeflexia and with positive pyramidal signs, (2) who experienced pain at the onset of the disease, excluding other potential causes including abscesses, tumors, infections, and trauma, (3) in addition to the symptoms of acute inflammatory polyneuropathy, urinary retention at the onset of symptoms and sensory level deficits are present [1, 8].

## Case presentation

A 17-year-old ambulating male with cerebral palsy presented to the ED with acute onset of bilateral symmetrical numbness and tingling starting in the feet that ascended rapidly within few hours to the legs with associated motor weakness and inability to ambulate. He had an episode of diarrhea preceding the onset of symptoms. The numbness progressed rapidly and halted once sensory deficits reached the level of T4. The patient complained of associated chest pain. The patient developed overflow incontinence due to urinary retention. Initial neurological examination was significant for bilateral lower limb motor weakness (grade 1/5), progressing to loss of sensation in both lower extremities with normal sensation above the nipple line. Deep and superficial reflexes gradually diminished over the next few hours.

**Figure 1:**
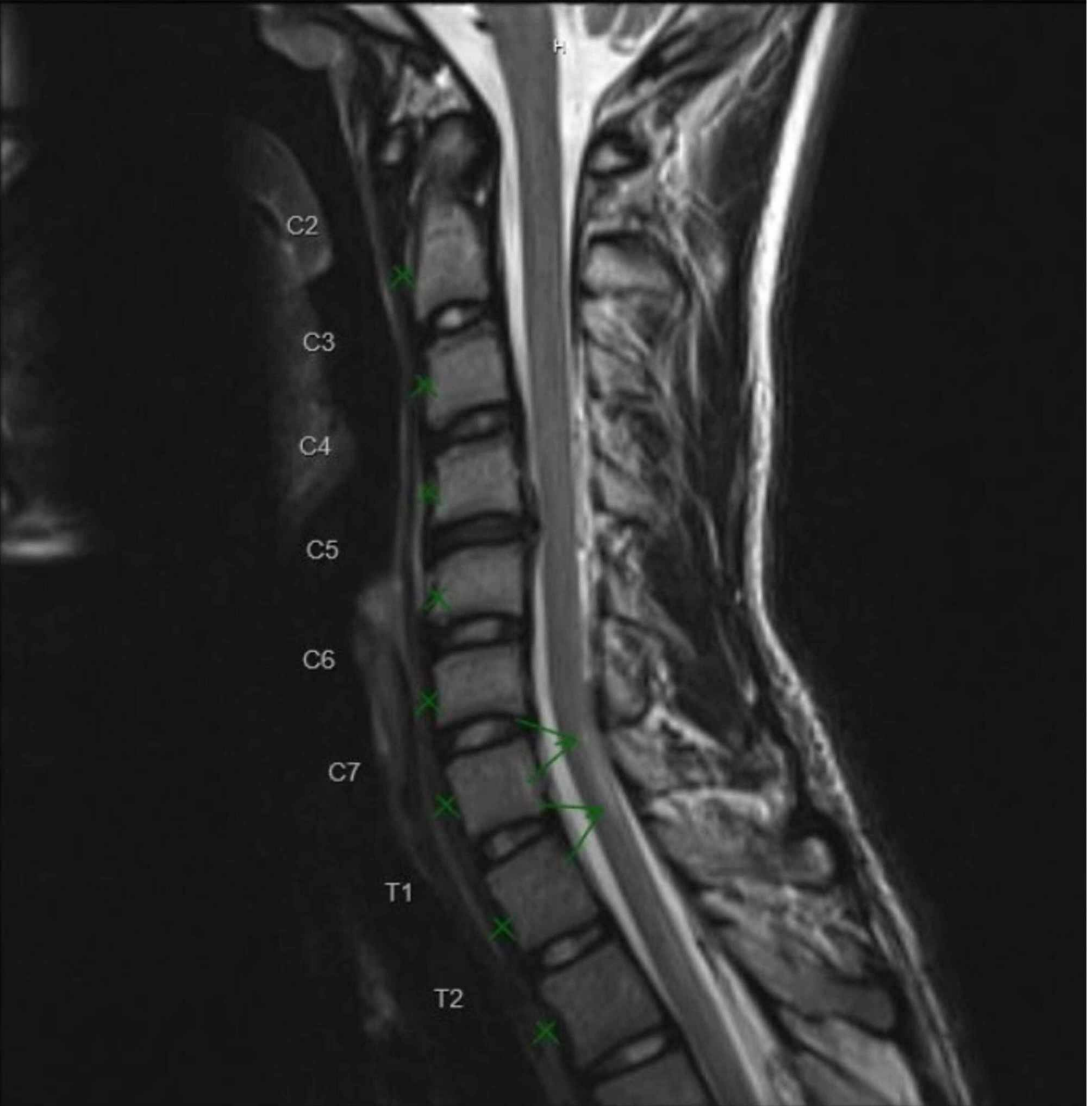
T2 weighted image of the cervical cord lesion. There is an increased T2 signal involving the dorsal aspect of the left side of the cord at the C7 level, extending over a length of approximately 1.5 cm (green arrows).

Cerebrospinal fluid (CSF) analysis showed albuminocytologic dissociation with 2 leukocytes/mm3, high protein content of 183 mg/dL (reference range 15-45 mg/dL), glucose level of 57 mg/dL (reference range 40-70 mg/dL), and negative bacterial cultures. The CSF albumin content was 83 mg/dL (reference range 0-35 mg/dL) and CSF IgG was elevated to 9.3 mg/dL (reference range 0.0-6.0 mg/dL). Blood immunology/serology, nonculture microbiology tests, and stool ova and parasite test to evaluate for infectious etiologies were negative. MRI of the spine revealed a lesion concerning for transverse myelitis at C7 level and high T2 intensity in the sacrum suggestive of inflammation (Figure 1).

The patient was treated with steroids (methylprednisolone 1 g daily for five days followed by steroid taper over two weeks) and intravenous immunoglobulin (IVIG) (0.4 g/kg daily for five days). After five days, significant improvement was noted in the motor weakness and sensation of the bilateral lower limbs. The bladder function was eventually regained. The patient was discharged to long-term rehabilitation.

## Discussion

The synchronous occurrence of GBS and ATM is not a common phenomenon. There are few cases in the published literature reporting this concurrence. 

Our patient presented with symptoms of GBS like numbness, paraesthesia, symmetric weakness, and hyporeflexia. The CSF findings supported our clinical findings. The rapid onset of symptoms, sensory level, bladder dysfunction, and MRI findings were indicative of spinal involvement. The nerve conduction study (NCS) as a diagnostic workup for the GBS could not be performed due to the limited capability of the hospital and constitutes a limitation in our case.

On reviewing the literature, review studies evaluating the cases of overlapping GBS and acquired demyelinating syndrome reported that most of the cases were preceded by infections or vaccinations [1, 9]. On the contrary, the etiology was unknown in our patient. In another retrospective review study, only a few cases were preceded by infections or vaccinations [10]. Such cases could indicate an immune response against epitopes that are common to components of both the central and the peripheral nervous system and are thus distinct from either ATM or GBS in isolation [11]. Further case reports may help to understand the pathophysiologic mechanism of this overlap.

As seen in our patient, the review studies also reported that these patients most often present with weakness, sensory deficits, urinary disturbance, sensory level, areflexia, or hyporeflexia [1, 9-10]. We believe that physicians should be aware of this association and include both diagnoses on differential when confronted with a patient presenting with these symptoms.

Most of the patients in these review studies had abnormalities in the spinal cord MRI. It can be concluded that spinal cord MRIs could be useful in the identification of ATM.

Treatment with IVIG is reported to be about as effective as plasma exchange in patients with the GBS [12]. Steroid therapy and plasma exchange are effective treatments for ATM [13-14]. Assuming that the pathogenesis of the spinal involvement is similar to that in ATM, a high-dosage course of pulsed corticosteroids can be considered as an effective treatment in these patients [15]. On the contrary, only 46% and 55% of the patients respectively, who received IVIG combined steroids had a favorable outcome [1, 9]. In the pediatric population, there is no level 1 evidence suggesting the treatment. Understanding pathophysiology and risk factors may help in providing appropriate treatment to these patients.

This work was previously presented as a poster at the 2020 Southern Regional Meeting and the abstract has been published in the *Journal of Investigative Medicine* [16].

## Conclusions

The differential should include simultaneous ATM and GBS in patients presenting with weakness, paresthesia, sensory deficits, urinary disturbance, sensory level, and areflexia or hyporeflexia. Considering the limited anecdotal evidence in the literature further may help to understand the pathophysiologic mechanisms underlying this synchronous occurrence and may provide better diagnostic and treatment approaches.

## References

[1] Guo F, Zhang YB (2019). Clinical features and prognosis of patients with Guillain-Barré and acute transverse myelitis overlap syndrome. Clin Neurol Neurosurg.

[2] Absoud M, Greenberg BM, Lim M, Lotze T, Thomas T, Deiva K (2016). Pediatric transverse myelitis. Neurology.

[3] Pidcock FS, Krishnan C, Crawford TO, Salorio CF, Trovato M, Kerr DA (2007). Acute transverse myelitis in childhood: center-based analysis of 47 cases. Neurology.

[4] Thomas T, Branson HM, Verhey LH (2012). The demographic, clinical, and magnetic resonance imaging (MRI) features of transverse myelitis in children. J Child Neurol.

[5] Knebusch M, Strassburg HM, Reiners K (1998). Acute transverse myelitis in childhood: nine cases and review of the literature. Dev Med Child Neurol.

[6] van den Berg B, Walgaard C, Drenthen J, Fokke C, Jacobs BC, van Doorn PA (2014). Guillain-Barré syndrome: pathogenesis, diagnosis, treatment and prognosis. Nat Rev Neurol.

[7] Hughes RA, Cornblath DR (2005). Guillain-Barré syndrome. Lancet.

[8] Oliveira LM, Cury RG, Castro LH, Nitrini R (2017). Concomitant transverse myelitis and acute axonal sensory-motor neuropathy in an elderly patient. Case Rep Immunol.

[9] Mao Z, Hu X (2014). Clinical characteristics and outcomes of patients with Guillain-Barré and acquired CNS demyelinating overlap syndrome: a cohort study based on a literature review. Neurol Res.

[10] Martens-Le Bouar H, Korinthenberg R (2002). Polyradiculoneuritis with myelitis: a rare differential diagnosis of Guillain-Barré syndrome. Neuropediatrics.

[11] Howell KB, Wanigasinghe J, Leventer RJ, Ryan MM (2007). Concomitant transverse myelitis and acute motor axonal neuropathy in an adolescent. Pediatr Neurol.

[12] Plasma Exchange/Sandoglobulin Guillain-Barré Syndrome Trial Group (1997). Randomised trial of plasma exchange, intravenous immunoglobulin, and combined treatments in Guillain-Barré syndrome. Lancet.

[13] Kaplin AI, Krishnan C, Deshpande DM, Pardo CA, Kerr DA (2005). Diagnosis and management of acute myelopathies. Neurologist.

[14] Defresne P, Hollenberg H, Husson B (2003). Acute transverse myelitis in children: clinical course and prognostic factors. J Child Neurol.

[15] Lahat E, Pillar G, Ravid S (1998). Rapid recovery from transverse myelopathy in children treated with methylprednisolone. Pediatric Neurol.

[16] (2020). 2020 Southern Regional Meeting. http://dx.doi.org/10.1136/jim-2020-SRM.

